# 

**Published:** 1892-06

**Authors:** 


					﻿Vol XII.
JUNE, 1892.
NO. 6?
THE
HOMEOPATHIC pHY^lClAp,
A Monthly Journal of Homoeopathic Materia Medica
and Clinical Medicine.
“ He it a freeman whom truth maker free, and all are tlavet betide."
"Seek the Truth: come whence U may, eott what it will."
EDITED AND H'BLISHID BT
WALTER M.‘ JAMES, M. D.
PHILADELPHIA :
No. lie© SPRUCE 8TREKT.
LO1TEON :
ALFRED HEATH A OOM Soli Aokmtb FOB Obbat Bbitaim,
114 Ebury Street, Eaton 8quare.
Yearly Sv been pt ion, $2.60. invariably in advance. Single number/, 26 etc
Entered at Ute Philadelphia Past-Ottos as Beeoed Class Mail Mataer.
				

